# Pro‐vitamin A carotenoids in East African highland banana and other *Musa* cultivars grown in Uganda

**DOI:** 10.1002/fsn3.1308

**Published:** 2019-12-09

**Authors:** Ruth Mbabazi, Robert Harding, Harjeet Khanna, Priver Namanya, Geofrey Arinaitwe, Wilberforce Tushemereirwe, James Dale, Jean‐Yves Paul

**Affiliations:** ^1^ National Agricultural Research Organisation, NARL Wakiso Uganda; ^2^ Centre for Tropical Crops and Biocommodities Queensland University of Technology Brisbane QLD Australia; ^3^ National Agricultural Research Organisation, National Coffee Research Institute Mukono Uganda; ^4^ Present address: Plant and Soil Science Building Michigan State University East Lansing MI USA; ^5^ Present address: Sugar Research Australia Indooroopilly QLD Australia

**Keywords:** banana, biofortification, carotenoids, micronutrient deficiency, pro‐vitamin A, vitamin A deficiency

## Abstract

Bananas and plantains (*Musa* spp.) are an important staple and food security crop in sub‐Saharan Africa. In Uganda, where the consumption of East African highland banana (EAHB) is the highest in the world, the population suffers from a high incidence of vitamin A deficiency (VAD). Since the consumption of pro‐vitamin A carotenoids (pVAC) made available through the food staple can help alleviate these ailments, we set out to identify the most suitable banana variety to use in future biofortification strategies through genetic engineering. The study focussed on eight popular *Musa* cultivars grown in the heart of banana farming communities and across the three major agricultural zones of Uganda. The fruit pVAC concentration varied considerably within and across the cultivars tested. These variations could not be explained by the altitude nor the geographical location where these fruits were grown. More than 50% of the total carotenoids present in EAHB cultivars was found to comprise of α‐ and β‐carotene, while the retention of these compounds following traditional processing methods was at least 70%. Storage up to 14 days postharvest improved carotenoid accumulation up to 2.4‐fold in the cultivar Nakitembe. The technical challenge for a successful biofortification approach in Uganda using genetically modified EAHB lies in guaranteeing that the fruit pVAC content will invariably provide at least 50% of the estimated average requirement for vitamin A regardless of the growing conditions.

## Graphical abstract

East African highland bananas (EAHBs) are the staple food of Uganda, a sub‐Saharan country where the incidence of vitamin A deficiency (VAD) is high. In addition to existing interventions, the biofortification of EAHBs with pro‐vitamin A carotenoids (pVAC) aims to help alleviate VAD, especially in subsistence farmer communities. In this study, fruits from banana varieties commonly grown in the region were analyzed for pVAC to identify the most suitable target for future biofortification efforts.

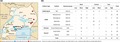

## 1 INTRODUCTION

Vitamin A deficiency (VAD) is an important public health problem in developing parts of Southeast Asia and sub‐Saharan Africa in particular with an estimated 250 million preschool children being affected (WHO, 2009). Although completely preventable through a balanced diet, VAD can lead to night blindness and other serious ailments such as keratomalacia and xerophthalmia often leading to deadly outcomes (Strobel, Tinz, & Biesalski 2007). VAD also greatly compromises the immune system thereby increasing the severity of common childhood infections such as measles and tropical diseases such as malaria (Rice, West, & Black, 2004).

Vitamin A cannot be synthesized de novo in the body. Therefore, humans rely on the dietary intake of animal‐sourced retinol and/or plant‐sourced pro‐vitamin A carotenoids (pVACs) for an adequate supply (Fitzpatrick et al., 2012). In parts of the world where populations cannot afford a diversified diet, staple crops such as rice, maize, sorghum, cassava, yam, taro, sweet potatoes, or banana constitute the bulk of the daily carbohydrate intake. Invariably, these staples are rich in starch but most are deficient in critical micronutrients such as pVACs and, as a result, a diet based on them leads to the exacerbation of VAD in these regions.

Bananas and plantains (*Musa* spp.) are important food staples for populations living in tropical and sub‐tropical areas of the globe. In the Great Lakes region of East Africa and in Uganda in particular, EAHB is the staple and constitutes the bulk of the diet of most rural and urban families (Adeniji, Tenkouano, Ezurike, Ariyo, & and, 2010). These bananas are traditionally harvested green, cooked and mashed into a local dish known as “matooke.” EAHBs are represented by more than one hundred farmer‐selected landrace cultivars all belonging to the Lujugira‐Mutika subgroup (AAA‐EA) (Kitavi et al., 2016). Agronomically and culturally accepted EAHB cultivars in East Africa have been reported to contain low levels of pVACs (Davey, Van den Bergh, Markham, Swennen, & Keulemans, 2009; Ekesa et al., 2013; Fungo & Pillay, 2011). Consequently, populations with an EAHB‐based diet are at increased risk of VAD. Accordingly, the 2006 Ugandan national demographic survey reported that the highest incidence of micronutrient deficiencies, including VAD, occurred in populations from regions where bananas are consumed as the principal source of carbohydrate (UDHS, 2006). As a result, in Uganda, 15 to 30% of children under the age of 60 months suffer from VAD with similar figures for women of childbearing age (UDHS, 2006).

Alleviating strategies such as supplementation and food fortification have been implemented in developing countries, including Uganda, over many years and have been demonstrably successful (Stevens et al., 2015). There remains, however, persistently high levels of VAD particularly in remote hard‐to‐reach areas that will only be addressed through more innovative strategies. Recently, biofortification has been implemented in an attempt to reduce micronutrient deficiencies among the “poorest of the poor” in a way that does not require constant external inputs (Bouis, 2003; Paine et al., 2005). By increasing the amount of essential nutrients in staple food crops, biofortification aims to target malnourished populations in a more economically viable and sustainable way. As such, where bananas are a major component of the daily carbohydrate intake, their biofortification with pVACs has the potential to help address VAD and therefore has become a key focus of local and international research and collaboration (Buah, Mlalazi, Khanna, Dale, & Mortimer, 2016; Paul et al., 2017).

Banana fruit carotenoid content is variable and highly cultivar dependent. The highest pVAC concentrations in banana were recorded from samples of the Micronesian Fe'i type banana “Utin Iap” collected in Pohnpei which ranged from 2,780 to 8,508 μg/100 g fresh weight (fw) β‐carotene (Englberger, Schierle, et al., 2006; Englberger, Schierle, Marks, & Fitzgerald, 2003). In contrast, the commercial Cavendish cultivar “Williams” has only 57 μg/100 g fw β‐carotene (Englberger, Wills, et al., 2006). Variations in fruit pVAC content have also been reported from west and central African banana and plantain cultivars (Davey et al., 2007, 2009) as well as EAHB cultivars, with amounts varying from 141 to 527 μg/100 g fw in cultivars “Kabucuragye” and “Nakitembe,” respectively (Fungo & Pillay, 2011). There is circumstantial evidence that both time of fruit development, and the prevailing environmental conditions plays a critical role in this pVAC variability (Paul et al., 2017).

The present study aimed to better understand carotenoid accumulation in the fruit of important *Musa* cultivars grown in Uganda with an emphasis on EAHBs. The baseline in carotenoid concentration was determined from mature green fruit of each cultivar using an extended sample size, and the levels of variations are reported. Considering the large diversity of agro‐ecological conditions in Uganda, the accumulation of carotenoids in banana fruit was also examined from various banana‐growing areas around the country to study the effects of changes in climatic conditions and altitudes. Finally, the influence of postharvest processes such as storage time, ripening and traditional cooking methods was investigated. The potential contribution of these cultivars to the estimated average requirement (EAR) for vitamin A is discussed in the context of biofortification.

## 2 MATERIALS AND METHODS

### 2.1 Banana cultivars and sample collection

Eight banana cultivars were included in this study which were representative of farmer‐ and consumer‐preferred bananas grown in Uganda. These included the plantain cultivar “Gonja Nakatansese” (*Musa* spp. AAB group), two dessert cultivars, “Bogoya” (*Musa* spp. AAA group) and “Sukali Ndiizi” (*Musa* spp. AAB group), four cooking‐type East African highland banana cultivars, “Mbwazirume,” “Mpologoma,” “Nakitembe,” and “Nakinyika” (EAHB ‐ *Musa* spp. AAA‐EA) and EAHB hybrid “M9” (Nowakunda et al., 2015).

**Figure 1:**
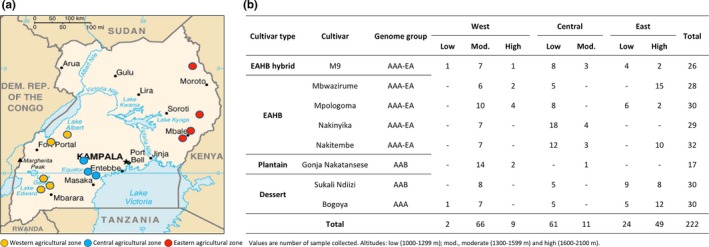
Banana sample collection in Uganda. (a) Location of banana sampling sites in the central, western, and eastern regions of Uganda and (b) numbers and origin of banana samples collected for this study

Bunches of fruit were harvested from plants growing in different locations in the western, central, and eastern regions of Uganda (Figure 1). In each region, bunches were taken from plants growing at low (1000–1299 m), moderate (1300–1599 m), and high (1600–2100 m) altitudes. All bunches were harvested at the full green (FG) mature stage and stored at room temperature in a well‐ventilated room before sampling and processing.

### 2.2 Banana sample processing and preparation

For all cultivars, a FG composite sample was made 1 day postharvest. This sample consisted of three fingers (one fruit from the top, middle, and bottom of each bunch). The peel was removed from each fruit and the pulp finely diced, homogenously mixed and the composite sample separated in three equal portions by weight. One portion was lyophilized immediately, ground to a fine powder using a mortar and pestle and stored at −80°C for subsequent carotenoid extraction and HPLC analysis. The other two portions (from EAHB and plantain cultivars only) were subjected to the traditional cooking method of steaming and boiling. For steaming, samples were wrapped in banana leaves which were placed in a saucepan on top of a charcoal stove for one hour. Samples processed by boiling were placed in 400 ml of boiling water for 15 min. Following processing, the weight of each sample was recorded and the fruit sample lyophilized, ground and stored at −80°C for subsequent analysis.

Composite samples were also made at day 7 and day 14 postharvest for all EAHB cultivars and at FG and full ripe (FR) for all dessert and plantain cultivars. These timeframes are representative of local storage and consumption habits. Harvested bunches were kept in the shade in a well‐aerated area and the fruit allowed to ripen naturally on the bunch. All fruits were picked from the same “hands” as the original FG samples.

### 2.3 Carotenoid extraction, quantification, and data analysis

Carotenoids were extracted from finely ground lyophilized banana fruit sample (200 mg) under low light conditions as previously described (Paul et al., 2017). Extracts were dried under vacuum and either analyzed immediately or stored at −20°C for up to 7 days. Carotenoid quantification by HPLC was done according to Buah et al. (2016) where extracts were auto‐injected into an Agilent 1,200 Series HPLC system equipped with a Multi‐Wavelength Detector (MWD) (Agilent Technologies) and carotenoids separated on a PrincetonSPHER C30 (5 μm, 4.6 × 250 mm) reverse‐phase column coupled to a 5 μm C30 guard cartridge (Princeton Chromatography Inc). All extracts were analyzed in two technical replicates. The amounts of the non‐pVAC lutein and the three pVACs, *trans*‐α‐carotene (*t*‐αC), *trans‐*β‐carotene (*t*‐βC), and *cis*‐β‐carotene(*c*‐βC) were measured according to Buah et al. (2016 **),** and total carotenoids, pVACs and β‐carotene equivalents (β‐CE) were calculated as follows; total carotenoids = lutein +t‐αC + *t*‐βC + *c*‐βC; pVAC = *t*‐αC + *t*‐βC + *c*‐βC and β‐CE = *t*‐βC + ½ (*t*‐αC + *c*‐βC). All calculations were expressed in μg per g on a dry weight basis (dw).

Statistical comparisons were made using the IBM SPSS Statistics Version 22 software package. Following Welch and Brown‐Forsythe tests of equality of means, mean differences were compared using either one‐way analysis of variance (ANOVA) and Least Significant Difference (LSD) post hoc test or nonparametric Kruskal–Wallis with statistical difference reported at 95% confidence level (*p* < .05).

## 3 RESULTS AND DISCUSSION

A total of 222 bunches were harvested from eight banana cultivars commonly grown across all major agricultural zones in the western, central, and eastern part of Uganda (Figure 1).

### 3.1 Fruit carotenoid content

**Figure 2:**
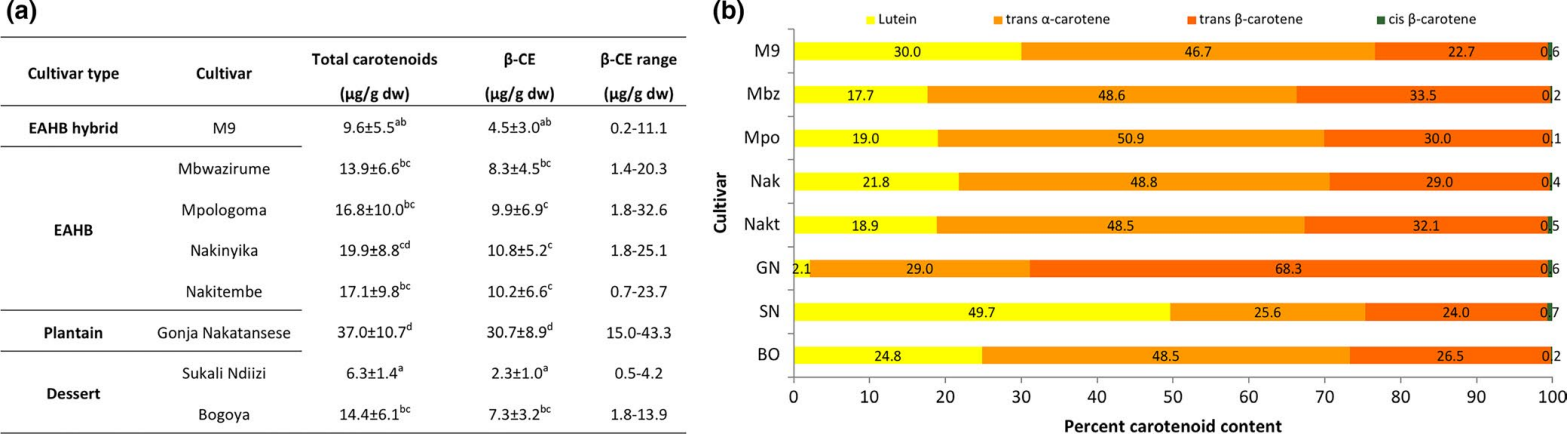
Carotenoid concentrations (a) and percentage accumulation of individual carotenoids (b) in the fruit pulp of mature green bananas from popular cultivars grown in Uganda. a, values are means ± *SD*. β‐CE = all‐trans β‐carotene equivalents. dw = dry weight. Statistical analysis comparing cultivars: Welch and Brown‐Forsythe tests of equality of means followed by independent samples nonparametric Kruskal–Wallis one‐way analysis of variance, different letters indicate significant difference at 95% confidence. b, percentage (%) carotenoid content calculated based on total carotenoid content in the fruit pulp. “M9” (*n* = 26), Mbz= “Mbwazirume” (*n* = 28), Mpo= “Mpologoma” (*n* = 30), Nak= “Nakinyika” (*n* = 29), Nakt= “Nakitembe” (*n* = 32), GN= “Gonja Nakatansese” (*n* = 17), SN= “Sukali Ndiizi” (*n* = 30), and BO= “Bogoya” (*n* = 30)

The baseline carotenoid concentration of each cultivar at harvest was determined by HPLC. A considerable amount of variability in the average amounts of total carotenoids (6.3 to 37.0 µg/g dry weight (dw)), β‐CE (2.3 to 30.7 µg/g dw), and pVACs (3.1 to 36.2 µg/g dw) was observed between the eight cultivars tested (Figure 2a; Table S1). Such inter‐cultivar variability in fruit pVAC content has been reported previously from the genus *Musa *(Davey et al., 2009). Using fruit from a very diverse set of banana cultivars grown under standardized conditions in Australia, Englberger, Wills, et al. (2006) reported β‐CE values from as low as 104 µg/100 g fresh weight (fw) (or 4.2 µg/g dw with the reported 75% water content) in the Cavendish cultivar “Williams,” to as high as 1593 µg/100 g fw (79.7 µg/g dw with the reported 80% water content) for the Fe'i banana “Asupina.” Studies using fruit from EAHBs have also shown a high degree of cultivar to cultivar variability in β‐CE levels, even when grown under similar conditions (Davey et al., 2009; Fungo & Pillay, 2011).

In the present study, fruit β‐CE levels from the plantain “Gonja Nakatansese” were statistically higher (*p* ≤ .01) than any other cultivar tested with decreasing amounts present in the four EAHBs “Nakinyika,” “Nakitembe,” “Mpologoma,” and “Mbwazirume,” followed by “Bogoya,” “M9,” and “Sukali Ndiizi” (Figure 2a). The high concentration of fruit pVAC in “Gonja Nakatansese” is consistent with reports on other plantain varieties (Davey et al., 2007, 2009; Newilah et al., 2008). Interestingly, there were no significant differences in fruit β‐CE levels between the four EAHB cultivars (*p *= 1) tested which were similar to those reported by Davey et al. (2009) for “Mbwazirume” and “Nakitembe.” Fruit carotenoids contents in the mainstream dessert Cavendish‐type (AAA) banana are universally low (Paul et al., 2017). However, our results from the cultivar “Bogoya” (AAA) were not significantly (*p* ≥ .3) different from “M9” nor any of the EAHBs (Figure 2a) while the small dessert banana “Sukali Ndiizi” had significantly lower (*p* < .01) fruit β‐CE levels than all other cultivar tested but hybrid “M9” (Figure 2a).

Of greater importance, it was observed that the mean fruit β‐CE content could vary by 29.1% in the plantain “Gonja Nakatansese” to up to 69.7% in the cultivars “Mpologoma” (Figure 2a). Similar levels of intracultivar variability have been previously reported in the plantains “Batard,” (8.6%) and Mbouroukou (46.6%) and in the Cavendish cultivar “Grande Naine” (57%) (Davey et al., 2007, 2009). In our study, these large variations could have been exacerbated by the diversity of sampling locations and agro‐ecological environments represented. Indeed, the lowest intracultivar biological variability came from “Gonja Nakatansese,” which also happened to have the narrowest agro‐ecological diversity of samples (Figure 1). Therefore, analysis of a larger pool of samples representing the largest agro‐ecological landscape in which these bananas are grown would be useful to determine the broader intracultivar variability. To determine the intracultivar stability in fruit pVAC content, however, samples should be collected from plants grown under the same conditions (Davey et al., 2007).

Despite the clear cultivar‐dependent genetic predisposition to metabolize and store fruit carotenoids, the intracultivar variation observed in the current study is likely to be influenced by variations in environmental conditions. Changes in temperature, light intensity, and rainfall have been implicated in influencing carotenoid accumulation in crops such as pumpkin (Jaswir, Shahidan, Othman, Has‐Yun Hashim, Octavianti, & Salleh 2014) carrot (Perrin et al., 2016) maize (Ortiz, Rocheford, & Ferruzzi, 2016) and banana (Davey et al., 2007; Paul et al., 2017). In a 3‐year field trial of the “Dwarf Cavendish” cultivar conducted in Australia, Paul et al. (2017) demonstrated that fruit β‐CE content varied between 1.0 and 8.1 µg/g dw depending on whether fruit was harvested in the summer or the winter months, respectively. The time taken from bunch emergence to harvesting a mature green fruit was a major factor influencing the accumulation of pVACs. Fruit from bunches that developed slowly in the winter months contained higher β‐CE levels than those that developed faster in the summer months. This direct relationship between longer bunch cycling time and increased fruit pVAC levels has been previously observed for many of the Fe'i bananas and the plantains. (Englberger, Schierle, et al., 2006) Further, EAHBs are also known for having a longer bunch maturation time than “Cavendish,” for example, and also accumulate more carotenoids in their fruit. In plantains, longer cycling times have been linked to the inherent low levels of gibberellins, possibly resulting from a diversion of flux down the carotenoid biosynthesis pathway (Davey et al., 2009; Lahav & Gottreich, 1984). The carotenoid biosynthesis pathway and the pathway leading to the production of gibberellins share the same precursor, geranylgeranyl pyrophosphate (GGPP), which is further converted into phytoene by the enzyme phytoene synthase (PSY). Whether a depletion in the GGPP pool is responsible for a similar phenomenon in other high‐pVA banana cultivars such as EAHBs and the Micronesian Fe'i banana is unknown.

Because of differences in chemical structures, the bioavailability and bioconversion of pVACs to vitamin A (retinol) in the body depends on the amounts and type of carotenoids present in the diet. In a complex diet, *trans*‐β‐carotene (*t‐*βC) is the most efficiently converted of all carotenoids (with a 12:1 bioconversion ratio to retinol) followed by *trans*‐α‐carotene (*t‐*αC, 6:1). (Yeum & Russell, 2002) Consequently, the relative proportions of individual carotenoids are important in a nutritional context. Analysis of the individual carotenoids present in the fruit of the cultivars in this study revealed that the main pVACs present were *t‐*αC, *t‐*βC, and *cis* β‐carotene (*c*‐βC) as well as the non‐pVAC, lutein (Figure 2b and Figure S1). On average, the four EAHB cultivars contained 19.4% lutein, 49.2% *t‐*αC, and only 31.2% *t‐*βC whereas the EAHB hybrid “M9” had higher amounts of lutein (30%) mainly to the detriment of *t‐*βC (22.7%), with only a 2.5% reduction in the amount of *t*‐αC (Figure 2b). The dessert cultivar “Bogoya” had a carotenoid profile very similar to other EAHB cultivars tested while “Sukali Ndiizi,” which was consistently low in pVACs, accumulated a large proportion of lutein (49.7%), and only 25.5% and 24% of *t‐*αC and *t‐*βC, respectively (Figure 2b). Our findings demonstrate that, except for the cultivar “Sukali Ndiizi” where the non‐pVAC lutein was predominant, *t‐*αC and *t‐*βC were most abundant in the other cultivars (on average >80% of total carotenoids). The elevated fruit β‐CE levels in the plantain “Gonja Nakatansese” are attributed to the large proportion of *t‐*βC (68.3%) and *t‐*αC (29%) (Figure 2b). In studies using other EAHBs (“Nshikazi” and “Vulambya”) and plantains (“Musheba” and “Musilongo”) from the eastern Democratic Republic of Congo (DRC), *t‐*βC and *t‐*αC were also found to constitute the bulk of all carotenoids found in the fruit (Ekesa et al., 2013). In fact, in most bananas, irrespective of the genome group, *t‐*βC and *t‐*αC combined have been reported to comprise more than 90% of all available pVACs in fruit and, in general, around 80% or more of the total amount of carotenoids (Davey et al., 2009; Ekesa et al., 2013; Englberger, Wills, et al., 2006). Interestingly, in our study, the percentage contribution of each carotenoid remained stable irrespective of the total pVAC levels measured in a particular fruit (Figure S1). This finding is also consistent with the results of Davey et al. (2009) and suggest a genotype‐specific predisposition for certain types of carotenoids. The simple carotenoid profile observed in most bananas appears to be characteristic of the genus *Musa*. Indeed, other plant species that have been extensively studied display a much more complex carotenoid profile including larger proportions of xanthophylls that have not been reported from the fruit of *Musa* spp. (Römer et al., 2000; Vallabhaneni et al., 2009).

The spatio‐temporal, developmental, and environmental stimuli‐dependent regulation of carotenoids in plants is very complex. The enzyme lycopene β‐cyclase catalyzes the formation of *t*‐βC by the addition of a β‐ring at each end of the precursor molecule lycopene (Botella‐Pavía & Rodríguez‐Concepción, 2006). In contrast, the formation of *t*‐αC from the same precursor is catalyzed by both the action of lycopene ε‐cyclase (adding an ε‐ring) and lycopene β‐cyclase (adding a β‐ring). Further downstream processing of *t*‐αC and *t*‐βC by hydroxylation at the C‐3 position of each ring produces the xanthophylls, zeaxanthin, and lutein, respectively. The substantial amounts of lutein detected (close to 50%) in “Sukali Ndiizi” in the study indicates a preference for the formation of αC at the cyclase branching point, probably due to an increase in lycopene ε‐cyclase activity and a subsequent high level of hydroxylation into lutein. This has important implications for biofortification. Indeed, although cyclases control the diversion of flux at the branching of the pathway, their actions do not necessarily result in enhanced accumulation of pVACs because of potential hydroxylation into non‐pVACs. Furthermore, the detection of lutein as the only xanthophyll in all cultivars tested indicates active β‐ and ε‐ring hydroxylases in banana fruit. Therefore, the hydroxylation of *t*‐βC by β‐ring hydroxylases should occur on the other branch of the pathway, producing at least zeaxanthin. Since lutein is the end product of the αC branching, it could be speculated that zeaxanthin and downstream xanthophylls are also being made in banana fruit but swiftly converted into downstream products such as the phytohormone, abscisic acid (ABA). This idea is supported by the critical role played by ABA in banana fruit development and its involvement in the initiation and progression of the ethylene‐mediated ripening process (Jiang, Joyce, & Macnish, 2000).

There has been only one study investigating the relationship between the expression of genes involved in carotenoid biosynthesis and carotenoid accumulation during fruit ripening in banana (Buah et al., 2016). When the expression levels of five genes (*DXS*, *GGPS*, *PSY1*, *PSY2a*, and *LCYB*) in the fruit of “Cavendish” (with low fruit pVAC) and “Asupina” (with high fruit pVAC) were compared, no meaningful correlation was observed to explain the biologically significant differences in fruit pVAC accumulation between the two cultivars. While gene expression did not correlate with fruit pVAC accumulation in banana (Buah et al., 2016; Paul et al., 2017) a body of evidence is accumulating to suggest that cellular structural changes occurring during fruit maturation and ripening could be responsible for the exponential accumulation of pVACs in certain type of bananas such as the Fe'i type, “Asupina.” For example, the cauliflower Orange gene (*Or*) mutation mediates the differentiation of proplastids into chromoplasts allowing a greater sink for carotenoid accumulation and high levels of β‐carotene accumulation in tissues normally devoid of carotenoids (Lu et al., 2006). OR also appears to act as an enhancer of carotenoid biosynthesis by post‐transcriptionally regulating phytoene synthase in *Arabidopsis *(Zhou et al., 2015). The presence of a functional homolog of *Or* in *Musa* has been suggested, especially in high pVAC Fe'i bananas such as “Asupina”; however, this remains to be demonstrated (Buah et al., 2016).

### 3.2 Effect of storage and ripening

In Uganda, EAHBs are typically purchased by the bunch which is stored until fully consumed. To investigate whether the composition of the fruit naturally changes through the ripening process during storage, the carotenoid concentration was measured in the fruit of the eight cultivars at 1 (FG), 7, and 14 days postharvest. In all EAHB cultivars tested, as well as “M9,” an increase in β‐CE was observed from the FG stage to day 14 postharvest (Table 1). This increase, however, was only significant for “Mbwazirume” (*p* = .049), “Mpologoma,” (*p* = .013) and “Nakitembe” (*p* < .01). Fruit from the cultivar “Nakitembe” had the greatest significant (*p* < .01) increase (2.4‐fold) in β‐CE during storage, and this was associated with significant (*p* < .01) increases in all pVACs measured (*t*‐αC, *t*‐βC and *c*‐βC) and also in the xanthophyll, lutein (*p* < .01) (Table S2). After 14 days of storage, “Nakitembe” accumulated almost 25 µg/g dw β‐CE in its fruit. Since “Nakitembe” is one of the preferred cultivars in Uganda, the consumption of these bananas could make a significant impact in delivering 50% of the EAR for vitamin A. (Davey et al., 2009; Paul et al., 2017) However, because of a number of logistical limitations in our study, only 3 biological replicates of this cultivar were analyzed. Therefore, further investigation using a larger number of samples is required to confirm this initial finding. Similar increases in pVACs have been recorded during the early stages of storage and ripening of the EAHBs “Nshikazi” and “Vulambya” from eastern DRC. (Ekesa et al., 2013) However, these increases were also followed by a significant decrease in pVAC levels as the fruit ripened beyond their normal consumption stage. A similar trend was also seen in fruit from the two plantains, “Musheba” and “Musilongo.” Unfortunately, our samples were not analyzed beyond day 14 for all EAHBs, and only at the full ripe (FR) stage for the plantain “Gonja Nakatansese.” In this cultivar, fruit β‐CE levels had reduced (around 30%) significantly (*p* = .01) by the FR stage and this was largely attributed to a significant (*p* < .01) reduction in the levels of *t*‐βC (Table 1 and S2). This trend is not only consistent with the study from Ekesa et al. (2013) but also with data from 10 locally‐grown plantain cultivars in Cameroon. (Newilah et al., 2009) Analysis of fruit from the dessert banana “Sukali Ndiizi” revealed a significant (*p* < .01) reduction in β‐CE during ripening as well as a significant (*p* < .01) increase in the levels of the non‐pVAC lutein (Table S2). Although an increase in fruit β‐CE concentration was seen in the other dessert cultivar “Bogoya,” this was not significant (*p* = .061).

**Table 1:** Effect of storage time and ripening on the carotenoid concentrations in the fruit of popular banana cultivars grown in Uganda

Cultivar type	Cultivar	Full green (FG)	Storage		Ripe	Fold change FG to Day 14
			Day 7	Day 14		
EAHB hybrid	M9	4.5 ± 3.0^a^	5.1 ± 0.4^a^	7.2 ± 1.6^a^	‐	1.6
EAHB	Mbwazirume	8.3 ± 4.5^a^	9.8 ± 1.3^ab^	13.1 ± 3.2^b^	‐	1.6
	Mpologoma	9.9 ± 6.9^a^	13.5 ± 1.1^b^	19.2 ± 2.3^c^	‐	1.9
	Nakinyika	10.8 ± 5.2^ab^	9.2 ± 0.2^a^	13.4 ± 1.1^b^	‐	1.2
	Nakitembe	10.2 ± 6.6^a^	15.4 ± 0.5^b^	24.9 ± 0.4^c^	‐	2.4
Plantain	Gonja Nakatansese	30.7 ± 8.9^a^	‐	‐	22.0 ± 9.5^b^	0.7
Dessert	Sukali Ndiizi	2.3 ± 1.0^a^	‐	‐	0.8 ± 0.3^b^	0.3
	Bogoya	7.3 ± 3.2^a^	‐	‐	8.8 ± 2.5^a^	1.2

Note

Values are mean all‐*trans* β‐carotene equivalents (μg/g dry weight) ± *SD*. Green sample: “M9” (*n* = 26), “Mbwazirume” (*n* = 28), “Mpologoma” (*n* = 30), “Nakinyika” (*n* = 29), “Nakitembe” (*n* = 32), “Gonja Nakatansese” (*n* = 17), “Sukali Ndiizi” (*n* = 30), and “Bogoya” (*n* = 30). For storage experiment at Day 7 and Day 14: *n* = 4 for all cultivars except “Nakitembe” for which *n* = 3. For ripening experiment: “Sukali Ndiizi” (*n* = 30), “Bogoya” (*n* = 30), and “Gonja Nakatansese” (*n* = 17). Statistical analysis comparing treatments: Levene's test for equality of variances followed by independent‐sample T‐test, different letters indicate significant difference at 95% confidence.

During storage and ripening, the matrix of the banana fruit pulp changes from hard and starch‐rich to soft and full of simple sugars. These physical changes might render stored carotenoids more readily available for extraction, thus explaining the higher β‐CE levels observed after 7 and 14 days of storage (for EAHB) and subsequently at the full ripe stage (for dessert bananas and the plantain). Other physiological changes such as the conversion of amyloplasts to chromoplasts also occur, potentially providing the fruit with temporary extra sink capacity for carotenoid storage (Buah et al., 2016).

### 3.3 Effect of traditional cooking methods

In Uganda, the traditional popular dish “matooke” is prepared from mature green EAHB fruit steamed while wrapped in banana leaves, or made from boiled fruit. Other cooking bananas, such as plantains, are roasted or deep fried. Exposure of various plant food material to similar conditions has been shown to influence carotenoid content in a highly variable manner (Shin, Heo, Seo, Choi, & Lee, 2016). Therefore, the retention of carotenoids in banana fruit following traditional Ugandan steaming and boiling practices was investigated.

In general, both treatments had a negative impact on the remaining levels of carotenoids in the cooked fruit with steaming having the greatest influence (Table 2). Retention levels after steaming ranged from 57.3% to 79.6% compared with 55.4 to 93.9% after boiling (Table 2). The most significant (*p* < .01) reduction in carotenoid concentration was seen in the fruit of the plantain “Gonja Nakatansese” where only 55.4% and 57.3% of the β‐CE levels were retained after boiling and steaming, respectively (Table 2). Although boiling lowered the β‐CE levels in the cooked fruit of all the other cultivars tested, this reduction was not significant (*p* > .05). Steaming, on the other hand, significantly reduced the carotenoid content in three EAHB cultivars tested, “Mbwazirume” (*p* < .01), “Mpologoma,” (*p* = .028) and “Nakinyika” (*p* = .036) while the reduction in “Nakitembe” and “M9” was not significant (*p* = .056 and 0.075*,* respectively).

**Table 2:** Effect of traditional cooking methods on the carotenoid concentrations in the FG fruit of popular cultivars in Uganda

Cultivar type	Cultivar	Full green (FG)	Cooking method	
			Boiling (retention)	Steaming (retention)
EAHB hybrid	M9	4.5 ± 3.0^a^	3.7 ± 1.6^a^ (82.2%)	3.5 ± 1.3^a^ (77.8%)
EAHB	Mbwazirume	8.3 ± 4.5^a^	6.8 ± 2.9^ab^ (81.9%)	5.9 ± 2.2^b^ (71.1%)
	Mpologoma	9.9 ± 6.9^a^	9.3 ± 4.3^ab^ (93.9%)	7.0 ± 3.2^b^ (70.7%)
	Nakinyika	10.8 ± 5.2^a^	8.8 ± 3.2^ab^ (81.5%)	8.6 ± 3.4^b^ (79.6%)
	Nakitembe	10.2 ± 6.6^a^	9.2 ± 4.7^a^ (90.2%)	7.7 ± 3.9^a^ (75.5%)
Plantain	Gonja Nakatansese	30.7 ± 8.9^a^	17.0 ± 4.3^b^ (55.4%)	17.6 ± 5.5^b^ (57.3%)

Note

Values are mean all‐*trans* β‐carotene equivalents (μg/g dry weight) ± *SD*. “M9” (*n* = 26), “Mbwazirume” (*n* = 28), “Mpologoma” (*n* = 30), “Nakinyika” (*n* = 29), “Nakitembe” (*n* = 32), and “Gonja Nakatansese” (*n* = 17). Statistical analysis comparing treatments: One‐way ANOVA and LSD post hoc test, different letters indicate significant differences at 95% confidence.

To investigate the effects of boiling and steaming on individual fruit carotenoids, the pVAC profiles of selected fruit samples were compared both pre‐ and post‐treatment (Table S3). For “Gonja Nakatansese,” a significant reduction (*p* < .001) in both *t*‐αC and *t*‐βC after both steaming and boiling was shown to be largely responsible for the overall reduction in β‐CE content (Table S3). Similarly, the amount of *t*‐αC and *t*‐βC detected in all other cooked bananas tested was found to be lower after both boiling and steaming thus having a great impact on the β‐CE level (Table S3). Interestingly, with the exception of “Nakinyika” and “Gonja Nakatansese,” the quantity of the non‐pVAC lutein measured in all cultivars was higher following boiling while steaming had no significant influence. Cooking practices such as boiling and steaming, but also roasting, oven drying or deep frying, have been reported to impact the retention (or recovery) and bioavailability of carotenoids in processed food. The nature of the impact (negative or positive) depends on multiple factors such as original concentration of carotenoids, processing time and temperature and the composition of the food matrix.

The retention is a measure of the levels of carotenoids remaining in the food following processing. There have been many studies investigating the retention of carotenoids in a multitude of food types, from vegetables to staple crops. For example, the retention of β‐carotene in mashed orange‐fleshed sweet potato following boiling was estimated at around 80% in the variety Resisto. (Jaarsveld, Marais, Harmse, Nestel, & Rodriguez‐Amaya, 2006) Later studies demonstrated significant variation in the carotenoid content and retention capabilities of orange‐fleshed sweet potato varieties with postboiling retention varying between 70% and 81%. (Bengtsson, Namutebi, Alminger, & Svanberg, 2008) In pVA‐biofortified maize, β‐CE retention above 100% following roasting and boiling have been reported. (Mugode et al., 2014) Cooking and other processing techniques can have important disruptive effect on the tissue matrix and lead to increased downstream carotenoids extractability. (Hof, Gartner, & Tiljburg, 1998; Lessin & Schwartz, 1997) Exposure to elevated temperature during steaming and boiling help the inactivation of carotene oxidizing enzymes as well as disrupting carotene‐protein complexes allowing a greater release of bound carotenoids and thus making them more readily extractable using solvents. As such, controlled processes described by Mugode et al. (2014) may have improved the matrix release and extraction of carotenoids and account for the retention value of over 100%. In the plantain cultivar “Batard,” around 70 to 72% retention in total carotenoids was reported following 30–40 min whole fruit boiling (Newilah et al., 2008) while our study demonstrate more than 45% loss in the plantain “Gonja Nakatansese” following a similar procedure (Table S3). Since our method involved slicing the fruit prior to boiling, the observed differences may simply be the result of the increased surface area and leaching from our samples. Another study examining retention of pVACs in EAHBs from the eastern DRC reported only 50% and 48.7% retention in cultivars “Vulambya” and “Nshikazi,” respectively, after boiling (Ekesa et al., 2013). Their study also included the plantain cultivars “Musheba,” which retained 79% of its original pVACs, and “Musilongo” from which the concentration of pVACs more than doubled following boiling. The large number of variables in these studies clearly preclude the establishment of any trend on the influence of a particular cooking method on the retention of carotenoids in food.

Measuring bioavailability is a way to determine the proportion of a compound (carotenoids in our case) able to enter the body to perform its intended function. Carotenoid accessibility and bioavailability are clearly improved by food processing (Ryan, O’Connell, O’Sullivan, Aherne, & O’Brien, 2008). In green bananas, the important amount of indigestible resistant starch is believed to limit carotenoid bioavailability (Bresnahan et al., 2012). Nevertheless, cooking of green banana has been shown to make a significant twofold improvement to their retinol bioefficacy in Mongolian gerbils (Bresnahan et al., 2012). Although traditional cooking of EAHB can reduce available pVAC by up to 30%, this loss could potentially be compensated by the potential gain in bioavailability following cooking.

### 3.4 Effect of geographical location and altitude

Although bananas and plantains are grown throughout Uganda, they are more abundant in the western, central, and eastern regions where banana consumption is the highest (Karamura, Karamura, & Tinzaara, 2012). To determine the influence of provenance and altitude on the accumulation of fruit carotenoids, the β‐CE content of FG fruit harvested from the eight cultivars growing in the three‐main banana‐growing areas of Uganda and from low, moderate, and high altitudes in these regions was compared. Overall, geographical location did not appear to be an important factor affecting banana fruit pVAC accumulation. Indeed, significant differences in β‐CE levels were only found in three instances; fruit from cultivars “M9” (*p* = .044) and “Sukali Ndiizi” (*p* = .026) obtained from eastern Uganda contained significantly lower β‐CE levels than fruit from the same cultivars growing in western and central Uganda, while fruit from the EAHB “Mbwazirume” obtained from central and eastern Uganda contained significantly (*p* < .001) lower β‐CE levels than fruit from the same cultivars growing in western Uganda (Table 3). Similarly, altitude was not an important factor influencing banana fruit pVAC accumulation. No significant difference in β‐CE levels was recorded in fruit growing at different altitudes with the exception of the cultivar “Nakitembe” which had significantly (*p* = .049) lower fruit β‐CE levels when grown at high altitudes compared with low and moderate altitudes (Table 3).

**Table 3:** Effect of geographical location and altitude on the carotenoid concentrations in the green fruit of popular banana cultivars grown in Uganda

Cultivar type	Cultivar	Agricultural zone			Altitude		
		West	Central	East	Low	Mod.	High
EAHB hybrid	M9	4.9 ± 2.8^a^	5.7 ± 3.2^a^	1.9 ± 1.0^b^	4.1 ± 3.1^a^	5.7 ± 2.9^a^	2.4 ± 0.8^a^
EAHB	Mbwazirume	12.3 ± 4.8^a^	9.2 ± 2.5^ab^	5.9 ± 3.1^b^	9.2 ± 2.5^a^	10.9 ± 4.1^a^	7.1 ± 4.8^a^
	Mpologoma	8.3 ± 5.7^a^	11.3 ± 9.9^a^	11.4 ± 5.5^a^	11.1 ± 7.9^a^	7.2 ± 6.2^a^	11.7 ± 4.9^a^
	Nakinyika	11.9 ± 3.0^a^	10.5 ± 5.8^a^	‐	10.0 ± 5.4^a^	‐	12.1 ± 5.0^a^
	Nakitembe	12.0 ± 7.3^a^	11.4 ± 6.0^a^	7.3 ± 6.6^a^	12.9 ± 5.3^a^	10.0 ± 7.2^ab^	7.3 ± 6.6^b^
Plantain	Gonja Nakatansese	30.7 ± 9.2^a^	31.2 ± 0.0^a^	‐	‐	32.0 ± 8.4^a^	20.7 ± 8.1^a^
Dessert	Sukali Ndiizi	1.8 ± 1.2^a^	1.5 ± 0.5^a^	2.7 ± 0.8^b^	2.3 ± 1.0^a^	1.8 ± 1.2^a^	2.7 ± 0.7^a^
	Bogoya	7.8 ± 2.5^a^	7.3 ± 3.9^a^	7.1 ± 3.5^a^	8.0 ± 3.4^a^	7.3 ± 2.3^a^	6.8 ± 3.7^a^

Note

Values are mean all‐*trans* β‐carotene equivalents (μg/g dry weight) ±*SD*. *n* ≥ 3 replicates. Altitudes: low (1000–1299 m); mod., moderate (1300–1599 m) and high (1600–2100 m). Statistical analysis across columns: One‐way ANOVA and LSD post hoc test, different letters indicate significant differences at 95% confidence.

### 3.5 Implications in the context of biofortification in Uganda

African plantains are traditionally consumed either roasted or fried. These processing techniques usually exacerbate carotenoid losses making plantains an unsuitable target crop for biofortification. In contrast, EAHBs with their excellent local consumer acceptance, good pVAC retention following processing and the compound effect of improved bioavailability postcooking, are very suitable for a biofortification intervention in Uganda. To better appreciate the potential of these banana cultivars in alleviating dietary VADs in Uganda, our results were compiled and converted into retinol activity equivalents (RAE) and summarized in a dietary context (Table 4). Based on the Nutrient Reference Values for Australia and New Zealand, the EAR for children up to 3 years of age is 210 μg RAE/day and 500 μg RAE/day for women (NHMRC/MoH, 2005). According to these standards and assuming a 250 and 500 g daily portion for children (below 3 years of age) and women, respectively, the percentage contribution of each cultivar to the EAR was calculated (Table 4). The interpretation of these results takes into consideration that a suitable biofortified staple crop should consistently provide at least 50% of the EAR. Carotenoids obtained from raw dessert bananas are not only present in low quantities but are also poorly bioavailable due to the lack of cooking (Bresnahan et al., 2012). Therefore, our calculation included a bioconversion ratio from β‐carotene to retinol of 12:1 for raw bananas and 6:1 for cooked fruit. Under our assumptions, a substantial consumption of raw “Sukali Ndiizi” would only provide 5.7% (250 g consumed) and 4.8% (500 g consumed) of the EAR for children and women, respectively. To meet dietary target levels with this cultivar, the bioavailability of carotenoids would have to be increased by around 10‐fold making this cultivar unsuitable for biofortification. Although only a threefold increase would be required for the other dessert banana, “Bogoya,” the consumption of such a large quantity of dessert fruit is unlikely in Uganda. The local preference lies in the cooked local dish “matooke” for which much larger quantities are consumed daily. Hypothetically, consumption of boiled/steamed plantains could contribute to more than 80% and 70% of the vitamin A EAR for children and women, respectively. However, because of their high dry matter content, plantains are not suitable for this practice and are traditionally consumed baked, roasted or deep fried; processes that are notoriously detrimental to carotenoids. The cultivar “M9” is an EAHB hybrid developed by NARO to address disease pressure in the lowland banana‐growing areas of Uganda where the fungal disease Black Sigatoka is a major constraint to banana production. As such, it is popular among farmers but certainly not the preferred choice of consumers because of its lighter colored pulp. Steamed “M9” fruit could address 17.4% of children's vitamin A requirement and 14.6% for women. With this in mind, only a threefold increase in pVACs in this cultivar would provide a sustainable source of vitamin A to cover 50% of the EAR of vulnerable populations in the lowlands of Uganda. Traditional landraces such as “Mbwazirume,” “Mpologoma,” “Nakinyika,” and “Nakitembe” are favored among Ugandans because their deeper yellow color and texture make them more suitable for preparing “matooke”. Steamed EAHBs such as “Nakinyika” could provide as much as 42.6% and 35.8% of the EAR of children and women, respectively. Although these values are very encouraging and are very close to the target of addressing 50% of the EAR, they have to be understood in a more practical context. Our calculations are based on average β‐CE levels which were shown to vary greatly. Therefore, the current technical challenge for the successful biofortification of EAHBs does not lie in randomly achieving the target but rather in ensuring that the fruit pVACs levels consistently provide 50% or more of the EAR, regardless of the growing conditions.

**Table 4:** Effect of cooking methods on retinol activity equivalent (RAE) from popular banana cultivars grown in Uganda and the Estimated Average Requirement for vitamin A

Cultivar type	Cultivar	β‐CE^a^ (μg/100 g fw)			RAE^b^ (μg/100 g fw)			% EAR^c^—Children (<3 yrs)—250 g			% EAR^c^—Woman—500 g		
		Green raw	Post‐boiling	Post‐steaming	Green raw	Post‐boiling	Post‐steaming	Green raw	Post‐boiling	Post‐steaming	Green raw	**Post‐boiling**	**Post‐steaming**
EAHB hybrid	M9	112.5	92.5	87.5	9.4	15.4	14.6	11.2	18.3	17.4	9.4	15.4	14.6
EAHB	Mbwazirume	207.5	169.9	147.5	17.3	28.3	24.6	20.6	33.7	29.3	17.3	28.3	24.6
	Mpologoma	247.5	232.4	175.0	20.6	38.7	29.2	24.6	46.1	34.7	20.6	38.7	29.2
	Nakinyika	270.0	220.1	214.9	22.5	36.7	35.8	26.8	43.7	42.6	22.5	36.7	35.8
	Nakitembe	255.0	230.0	192.5	21.3	38.3	32.1	25.3	45.6	38.2	21.3	38.3	32.1
Plantain	Gonja Nakatansese	767.5	425.2	439.8	64.0	70.9	73.3	**76.1**	**84.4**	**87.3**	**64.0**	**70.9**	**73.3**
Dessert	Sukali Ndiizi	57.5	‐	‐	4.8	‐	‐	5.7	‐	‐	4.8	‐	‐
	Bogoya	182.5	‐	‐	15.2	‐	‐	18.1	‐	‐	15.2	‐	‐

a β‐carotene equivalents (β‐CE). Values estimated based on β‐CE data presented in Figure 2, A and assuming 25% dry matter content. “M9” (*n* = 26), “Mbwazirume” (*n* = 28), “Mpologoma” (*n* = 30), “Nakinyika” (*n* = 29), “Nakitembe” (*n* = 32), “Gonja Nakatansese” (*n* = 17), “Sukali Ndiizi” (*n* = 30), and “Bogoya” (*n* = 30). fw = fresh weight.

b Retinol activity equivalents (RAE) ‐ conversion factor from β‐carotene equivalents to RAE for raw banana was 12:1 and 6:1 for cooked banana. Conservative values based on a study by Bresnahan et al. (2012).

c Estimated Average Requirement (EAR) is 210 μg RAE/day for children 1–3 years old and 500 μg RAE/day for woman (Nutrient Reference Values for Australia and New Zealand, 2005).

Values in bold exceed the Banana 21 project target which is to achieve at least 50% of the vitamin A EAR.

## CONFLICTS OF INTEREST

The authors declare having no conflict of interest.

## ETHICAL APPROVAL

This study did not involve any human or animal testing.
