# Supplementary material for: Pro‐vitamin A carotenoids in East African highland banana and other Musa cultivars grown in Uganda
Source: Food Sci Nutr. 2019 Dec 9;8(1):311–21. doi: 10.1002/fsn3.1308 (PMC6977416; doi:10.1002/fsn3.1308)
Supplement: Supplementary file 1 [file FSN3-8-311-s001.pdf]

Supplementary information

Table S1. Carotenoid concentrations in the fruit pulp of green bananas from popular cultivars grown in Uganda

Cultivar type	Cultivar	Carotenoids (µg/g dw)				
		**Lutein**	***t*-αC**	***t*-βC**	***c*-βC**	**pVAC**
**EAHB hybrid**	M9	2.7±1.2^{abc}	4.7±3.3^{ab}	2.2±1.4^{ab}	0.1±0.1^{ab}	6.9±4.6^{ab}
**EAHB**	Mbwazirume	2.1±0.7^{a}	6.9±3.4^{bc}	4.9±2.9^{c}	0^{ab}	11.8±6.2^{bc}
	Mpologoma	2.4±0.5^{ab}	9.0±6.0^{bc}	5.4±4.1^{c}	0^{a}	14.5±9.9^{bc}
	Nakinyika	4.0±1.6^{c}	10.0±5.0^{c}	5.8±2.8^{c}	0.1±0.1^{ac}	15.8±7.7^{c}
	Nakitembe	2.4±0.9^{ab}	8.9±5.6^{bc}	5.7±3.9^{c}	0.1±0.1^{bc}	14.7±9.3^{c}
**Plantain**	Gonja Nakatansese	0.8±0.5^{d}	10.8±3.4^{c}	25.2±7.3^{d}	0.2±0.2^{c}	36.2±10.6^{d}
**Dessert**	Sukali Ndiizi	3.2±1.6^{abc}	1.6±0.8^{a}	1.5±0.7^{a}	0^{ab}	3.1±1.4^{a}
	Bogoya	3.4±1.5^{bc}	7.3±3.8^{bc}	3.7±1.4^{bc}	0^{a}	11±5.1^{bc}

Values are mean (µg/g dw) ± SD. dw= dry weight. *t*-αC= *trans*-α-carotene, *t*-βC= *trans*-β-carotene, *c*-βC= *cis*-β-carotene, pVAC= pro-vitamin A carotenoid. Statistical analysis comparing cultivars: nonparametric Kruskal-Wallis one-way analysis of variance, different letters indicate significant difference at 95% confidence. ‘M9’ (n= 26), ‘Mbwazirume’ (n= 28), ‘Mpologoma’ (n= 30), ‘Nakinyika’ (n= 29), ‘Nakitembe’ (n= 32), ‘Gonja Nakatansese’ (n= 17), ‘Sukali Ndiizi’ (n= 30) and ‘Bogoya’ (n= 30).

Table S2. Effect of traditional cooking methods on the concentration of important carotenoids in the fruit of EAHB and plantain cultivars

Cultivar	Sample	Carotenoids (µg/g dw)					
		Lutein	*t*-αC	*t*-βC	*c*-βC	Total carotenoids	pVAC
M9	FG	2.7±1.2[a]	4.7±3.3[a]	2.2±1.4[a]	0.1±0.1[a]	9.6±5.5[a]	6.9±4.6[a]
	Day 7	2.1±0.1[a]	4.6±0.2[a]	2.0±0.3[a]	0.8±0.1[b]	9.5±0.2[a]	7.4±0.3[a]
	Day 14	2.9±1.2[a]	6.9±1.7[a]	2.9±0.6[a]	0.9±0.2[b]	13.6±3.6[a]	10.7±2.4[a]
Mbwazirume	FG	2.1±0.7[a]	6.9±3.4[ab]	4.9±2.9[a]	-	13.9±6.6[a]	11.8±6.2[a]
	Day 7	2.0±0.3[a]	5.6±1.0[a]	4.4±0.5[a]	2.6±0.7[a]	14.5±1.9[a]	12.6±1.6[a]
	Day 14	2.1±0.2[a]	7.7±0.6[b]	5.7±1.6[a]	3.5±1.4[a]	19.0±3.4[a]	17.0±3.5[a]
Mpologoma	FG	2.4±0.5[a]	9.0±6.0[a]	5.4±4.1[ab]	-	16.8±10.0[a]	14.5±9.9[a]
	Day 7	3.2±0.2[b]	11.2±0.8[a]	6.3±0.7[a]	1.5±0.2[a]	22.2±1.3[b]	19.1±1.5[b]
	Day 14	2.4±0.1[a]	15.8±1.5[b]	9.6±2.3[b]	1.6±0.4[a]	22.6±1.9[c]	27.1±2.0[c]
Nakinyika	FG	4.0±1.6[a]	10.0±5.0[a]	5.8±2.8[a]	0.1±0.1[a]	19.9±8.8[a]	15.8±7.7[ab]
	Day 7	4.2±0.1[a]	8.0±0.2[b]	3.9±0.1[b]	1.3±0.0[b]	17.4±0.3[a]	13.2±0.2[a]
	Day 14	3.9±1.5[a]	11.5±2.4[ab]	5.8±0.3[a]	1.8±0.2[c]	23.3±3.9[a]	19.1±2.3[b]
Nakitembe	FG	2.4±0.9[a]	8.9±5.6[a]	5.7±3.9[a]	0.1±0.1[a]	17.1±9.8[a]	14.7±9.3[a]
	Day 7	3.5±0.2[b]	12.4±0.2[b]	7.5±0.3[b]	1.7±0.1[b]	25.0±0.7[b]	21.6±0.6[b]
	Day 14	4.0±0.8[b]	18.5±0.9[c]	13.2±0.7[c]	2.4±0.1[c]	38.0±0.8[c]	34.1±0.4[c]
Gonja Nakatansese	Full green	0.8±0.5[a]	10.8±3.4[a]	25.2±7.3[a]	0.2±0.2[a]	37.0±10.7[a]	36.2±10.6[a]
	Ripe	2.7±0.9[b]	8.7±3.5[a]	17.5±7.8[b]	0.3±0.2[a]	29.2±10.7[b]	26.5±11.2[b]
Sukali Ndiizi	Full green	3.2±1.6[a]	1.6±0.8[a]	1.5±0.7[a]	-	6.3±1.4[a]	3.1±1.4[a]
	Ripe	7.3±1.3[b]	0.6±0.4[b]	0.5±0.1[b]	-	8.3±1.6[b]	1.1±0.6[b]
Bogoya	Full green	3.4±1.4[a]	7.3±3.8[a]	3.7±1.4[a]	-	14.4±6.1[a]	11.0±5.1[a]
	Ripe	4.7±1.6[b]	8.4±3.1[a]	4.5±1.1[b]	0.1±0.2	17.7±4.8[b]	13.0±4.0[a]

Values are mean (µg/g dw) ± SD. dw= dry weight. *t*-αC= *trans*-α-carotene, *t*-βC= *trans*-β-carotene, *c*-βC= *cis*-β-carotene, pVAC= pro-vitamin A carotenoid. Green sample: 'M9' (n= 26), 'Mbwazirume' (n= 28), 'Mpologoma' (n= 30), 'Nakinyika' (n= 29), 'Nakitembe' (n= 32), 'Gonja Nakatansese' (n= 17), 'Sukali Ndiizi' (n= 30) and 'Bogoya' (n= 30). For storage experiment at Day 7 and Day 14: n= 4 for all cultivars except 'Nakitembe' for which n= 3. For ripening experiment: 'Sukali Ndiizi' (n= 30), 'Bogoya' (n= 30) and 'Gonja Nakatansese' (n= 17). Statistical analysis comparing treatments: Levene's test for equality of variances followed by independent-sample T-test, different letters indicate significant difference at 95% confidence.

Table S3. Effect of traditional cooking methods on the concentration of important carotenoids in the fruit of EAHB and plantain cultivars

Cultivar type	Cultivar	Sample	Carotenoids (µg/g dw)					
			Lutein	*t*-αC	*t*-βC	*c*-βC	Total carotenoids	pVAC
**EAHB hybrid**	M9	Full green	2.7±1.2[a]	4.7±3.3[a]	2.2±1.4[a]	0.1±0.1[a]	9.6±5.5[a]	6.9±4.6[a]
		Boiling	3.7±0.9[b]	3.7±1.7[a]	1.7±0.7[a]	0.1±0.1[a]	9.2±3.0[a]	5.6±2.4[a]
		Steaming	2.7±0.5[a]	3.5±1.4[a]	1.7±0.6[a]	0.2±0.1[b]	8.1±2.2[a]	5.3±2.0[a]
**EAHB**	Mbwazirume	Full green	2.1±0.7[a]	6.9±3.4[a]	4.9±2.9[a]	-	13.9±6.6[a]	11.8±6.2[a]
		Boiling	2.6±0.4[b]	5.6±2.1[ab]	3.9±1.9[ab]	0.2±0.1[a]	12.2±4.0[ab]	9.6±4.0[ab]
		Steaming	2.2±0.4[a]	4.8±1.6[b]	3.3±1.4[b]	0.4±0.2[b]	10.7±3.1[b]	8.5±3.1[b]
	Mpologoma	Full green	2.4±0.5[a]	9.0±6.0[a]	5.4±4.1[a]	-	16.8±10.0[a]	14.5±9.9[a]
		Boiling	4.0±0.9[b]	8.4±4.2[a]	5.1±2.3[ab]	0.1±0.1[a]	17.5±6.3[a]	13.6±6.4[ab]
		Steaming	2.6±0.6[a]	5.9±2.7[b]	3.8±1.8[b]	0.5±0.3[b]	12.8±4.4[b]	10.3±4.5[b]
	Nakinyika	Full green	4.0±1.6[a]	10.0±5.0[a]	5.8±2.8[a]	0.1±0.1[a]	19.9±8.8[a]	15.8±7.7[a]
		Boiling	4.0±1.0[a]	7.9±3.1[b]	4.7±1.7[ab]	0.3±0.2[b]	16.9±5.4[ab]	12.9±4.8[a]
		Steaming	3.4±1.0[b]	7.9±3.5[b]	4.4±1.7[b]	0.4±0.2[c]	16.1±5.7[b]	12.7±5.1[a]
	Nakitembe	Full green	2.4±0.9[a]	8.9±5.6[a]	5.7±3.9[a]	0.1±0.1[a]	17.1±9.8[a]	14.7±9.3[a]
		Boiling	3.5±1.0[b]	7.7±3.9[ab]	5.2±2.8[a]	0.2±0.1[b]	16.6±6.8[ab]	13.1±6.7[ab]
		Steaming	2.0±0.9[a]	6.2±3.0[b]	4.4±2.5[a]	0.3±0.2[c]	13.0±5.4[b]	11.0±5.4[b]
**Plantain**	Gonja Nakatansese	Full green	0.7±0.5[a]	10.8±3.4[a]	25.2±7.3[a]	0.2±0.2[a]	37.0±10.7[a]	36.2±10.6[a]
		Boiling	0.6±0.3[a]	4.8±1.9[b]	14.4±3.6[b]	0.4±0.2[a]	20.2±5.2[b]	19.6±5.1[b]
		Steaming	0.5±0.5[a]	5.1±2.6[b]	14.5±4.8[b]	1.1±0.7[b]	21.2±6.3[b]	20.6±6.4[b]

Values are mean (µg/g dw) ± SD. dw= dry weight. *t*-αC= *trans*-α-carotene, *t*-βC= *trans*-β-carotene, *c*-βC= *cis*-β-carotene, pVAC= pro-vitamin A carotenoid. 'M9' (n= 26), 'Mbwazirume' (n= 28), 'Mpologoma' (n= 30), 'Nakinyika' (n= 29), 'Nakitembe' (n= 32) and 'Gonja Nakatansese' (n= 17). DW, dry weight. Statistical analysis per columns: One-way ANOVA and LSD post-hoc test, different letters indicate significant differences at 95% confidence.

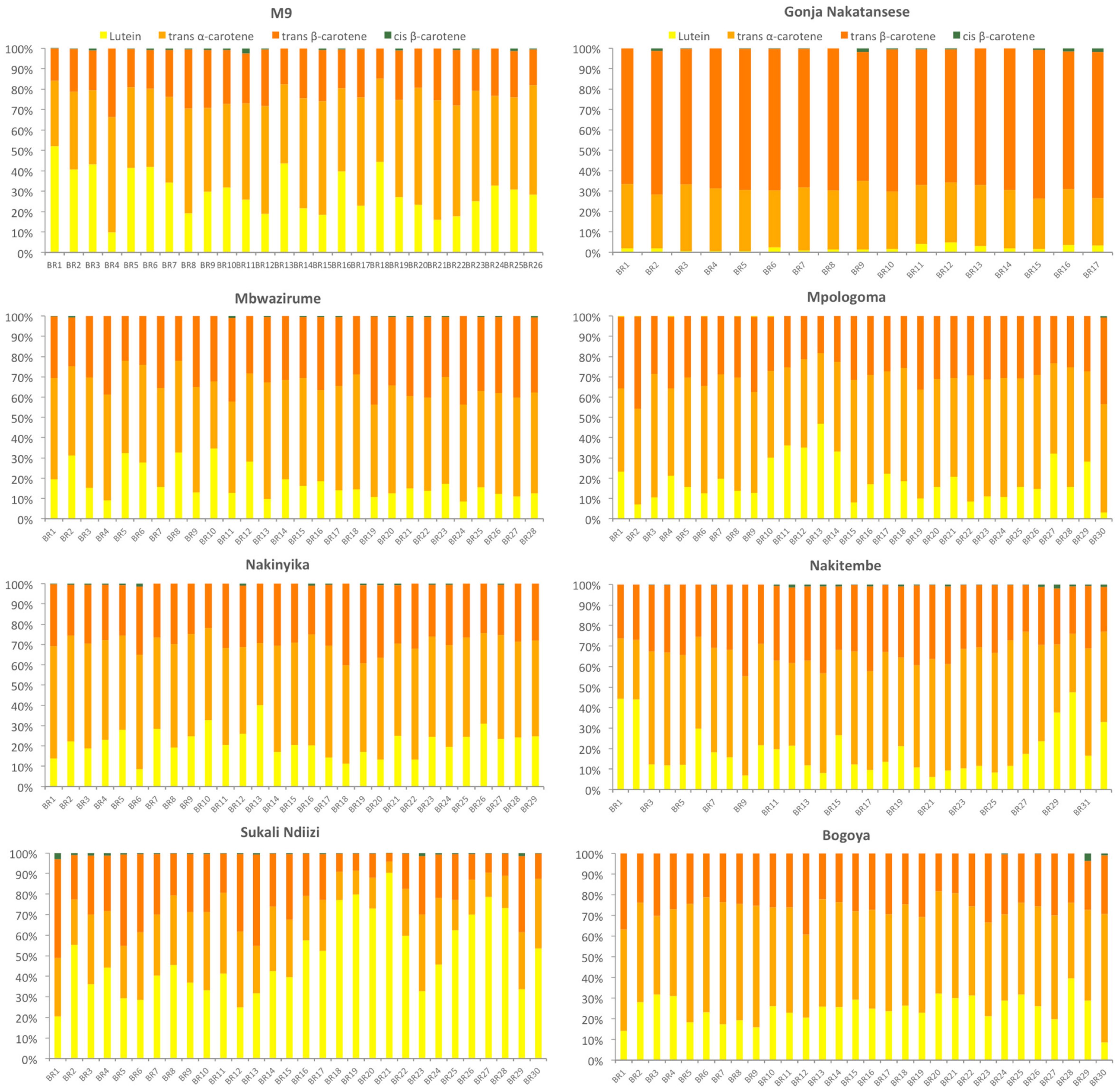

Figure S1. Percentage accumulation of individual carotenoids in the fruit pulp of mature full green (FG) bananas for each individual samples tested for each cultivars. Data represent percentage (%) carotenoid content calculated based on total carotenoid content in the fruit pulp. 'M9' (n= 26), Mbz= 'Mbwazirume' (n= 28), Mpo= 'Mpologoma' (n= 30), Nak= 'Nakinyika' (n= 29), Nakt= 'Nakitembe' (n= 32), GN= 'Gonja Nakatansese' (n= 17), SN= 'Sukali Ndiizi' (n= 30) and BO= 'Bogoya' (n= 30).
